# Biological Potential of Chitinolytic Marine Bacteria

**DOI:** 10.3390/md14120230

**Published:** 2016-12-16

**Authors:** Sara Skøtt Paulsen, Birgitte Andersen, Lone Gram, Henrique Machado

**Affiliations:** 1 Department of Biotechnology and Biomedicine, Technical University of Denmark, DK-2800 Kgs. Lyngby, Denmark; saskp@dtu.dk (S.S.P.); ba@bio.dtu.dk (B.A.); henma@biosustain.dtu.dk (H.M.); 2 Novo Nordisk Foundation Center for Biosustainability, Technical University of Denmark, DK-2800 Kgs. Lyngby, Denmark

**Keywords:** chitin, chitinases, antifungal, marine bacteria, *Pseudoalteromonadaceae*, *Vibrionaceae*

## Abstract

Chitinolytic microorganisms secrete a range of chitin modifying enzymes, which can be exploited for production of chitin derived products or as fungal or pest control agents. Here, we explored the potential of 11 marine bacteria (*Pseudoalteromonadaceae*, *Vibrionaceae*) for chitin degradation using in silico and phenotypic assays. Of 10 chitinolytic strains, three strains, *Photobacterium galatheae* S2753, *Pseudoalteromonas piscicida* S2040 and S2724, produced large clearing zones on chitin plates. All strains were antifungal, but against different fungal targets. One strain, *Pseudoalteromonas piscicida* S2040, had a pronounced antifungal activity against all seven fungal strains. There was no correlation between the number of chitin modifying enzymes as found by genome mining and the chitin degrading activity as measured by size of clearing zones on chitin agar. Based on in silico and in vitro analyses, we cloned and expressed two ChiA-like chitinases from the two most potent candidates to exemplify the industrial potential.

## 1. Introduction

Chitin, the β-1,4-linked homopolymer of *N*-acetylglucosamine (GlcNAc), is the most abundant polymer in the marine environment, and the second in nature after cellulose. Chitin is the structural basis for exoskeletons of crustaceans and insects, and a component of the fungal cell wall. The global production of chitin is estimated to be 10^11^ tons per year, however, chitin does not accumulate as it is hydrolyzed by marine microorganisms [1,2]. The hydrolysis is mediated by chitinolytic enzymes and allows the microorganisms to utilize chitin as a carbon and nitrogen source and chitin turnover is important for the biogeochemical C- and N-cycles. Chitin and chitinolytic enzymes are also of biotechnological interest with potential applications in the food, medical and agricultural sectors [3]. Also, chitin in the form of shellfish waste can be considered as a resource potentially used as a carbon-source in microbial fermentations. Shellfish waste constitutes an environmental problem of increasing magnitude [4,5], and the discovery of inexpensive processes, which can degrade chitin into chitooligosaccharides, chitosan and GlcNAc, may address this problem [6].

As mentioned, the cell wall of fungi contains chitin and some chitinolytic microorganisms can inhibit the growth of fungi by chitin degradation [7,8,9]. Fungal plant diseases are of great concern in agriculture and cause large losses at an estimated 5%–10% of the world’s food production [10]. Fungal contamination and mycotoxin production is also a problem in the built environment [11]. Potentially, natural fungicides, such as chitinases, could replace the chemical fungicides in plant biocontrol [12] and toxic chemicals indoors, and since bacterial chitinases can inhibit fungal growth, they are of particular interest for this purpose [7,13].

Chitin is a recalcitrant insoluble polysaccharide and is degraded into soluble oligosaccharides or GlcNAc. Chitinases (EC 3.2.1.14) hydrolyze the β-1,4 glycosidic bonds between the GlcNAc residues to produce chitooligosaccharides. Chitinases are glycosyl hydrolases (GH) and are divided into GH families 18 and 19. Bacterial chitinases usually belong to family 18, although a few belonging to family 19 have been described [14]. The GH18 and GH19 chitinases differ in sequence similarity, three-dimensional structure and catalytic mechanism. Bacteria often secrete many chitinases, and it is believed that they do so in order to efficiently hydrolyze the different forms of chitin they encounter [7,15,16]. The GH19 chitinases are believed to be the primary enzymes involved in breakdown of fungal chitin, but this is also mediated by other types of chitinases [7,17].

Recently, a new extracellular enzyme involved in breakdown of chitin was discovered. Lytic polysaccharide monooxygenases (LPMOs) were first described in 2010 [18] and are metalloenzymes that oxidize the glycosidic bonds in the crystalline surface of chitin and facilitate access of chitinases. LPMOs were first classified as carbohydrate binding module family 33 and they were believed only to be involved in substrate recognition [19]. LPMOs are now reclassified in auxiliary activity group 10 (AA10) in the CAZY-database [20] and their facilitating activity in chitin degradation has become clear [21]. Another facilitating enzyme, which has so far only been described in some marine bacteria, is chitooligosaccharide deacetylase (COD) [22]. COD (EC 3.5.1.105) is secreted in low concentrations and produces a signal molecule, GlcNac-GlcN, which acts as an inducer for chitinase production [23]. Deacetylation by COD is of particular interest for the industrial production of chitosan oligomers. A combination of COD and another chitin deacetylase, NodB, is used for commercial production of defined chitosan oligomers [24].

The purpose of the present work was to determine the potential for chitin degradation in a collection of marine bacteria. The bacteria were isolated due to their production of antibacterial compounds [25] and we have recently shown that chitin influences the production of secondary metabolites, such as antibacterial compounds, in some of these bacteria [26,27]. We therefore rationalized that they likely would have a high potential for chitin degradation. We envision that these bacteria and their chitinolytic activities will be of interest to the biotech and building industries and in agricultural production. We used in silico genome-wide analysis combined with phenotypic testing to unravel the potential and to exemplify the industrial perspective we cloned and heterologously expressed two chitinases.

## 2. Results

### 2.1. Chitin Degrading Activity and in Silico Analysis

Chitin degradation by 11 bacterial strains was determined on agar plates containing crystalline chitin, colloidal chitin or chitosan of both shrimp and crab origin. All but one strain, *P. fuliginea* S3431, degraded chitin. At low temperatures (4 °C and 15 °C), the clearing zones in the chitin containing media were hardly visible. The most pronounced chitin hydrolysis was seen at 35 °C and 25 °C. There was no particular difference between degradation of crab and shrimp chitin. Degradation of crystalline chitin was slower than that of colloidal chitin. None of the strains had chitosanase activity.

The bacterial genomes have previously been mined for chitinase genes and ChiS [28], and we here extend this genome mining including also COD, LPMO and a subdivision of the chitinase genes (Table 1). Two to six putative chitinase genes were found in the 10 chitin degrading strains, and all but one strain, *V. galatheae* S2757, also encoded one to two putative LPMOs. The gene encoding the ChiS sensor was only present in strains belonging to the *Vibrionaceae* family, whereas the CdsS/CdsR pair was present in all *Pseudoalteromonas* strains, including strain S3431 that did not degrade chitin.

We analysed the chitinases in Pfam, NCBI and SignalP which enabled the classification of chitinases into GH18 and GH19 groups (Table 1). The GH18 chitinases were sub-divided into ChiA, ChiD and an unspecified group. GH18 and, interestingly, also GH19 chitinases were found in all strains, except S3431.

**Figure 1:**
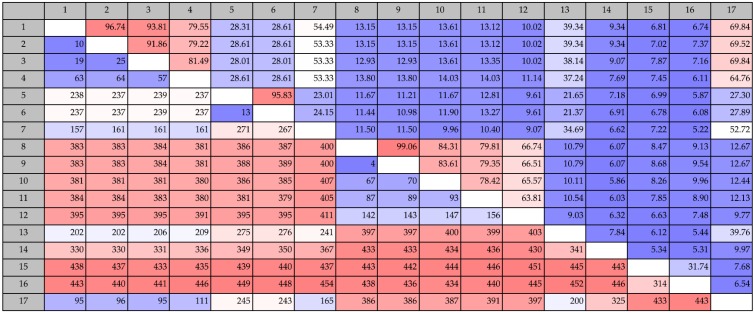
Pairwise comparison of protein sequence identities of putative COD genes from this study (numbers 1: *Vibrio coralliilyticus* (KJY71281), 2: *Vibrio neptunius* S2394 (KJY93856), 3: *Vibrio galatheae* S2757 (KJY81897), 4: *Vibrio nigripulchritudo* S2604 (KJY75235), 5: *Pseudoalteromonas piscicida* S2040 (KJY92988) and 6: *Pseudoalteromonas piscicida* S2724 (KJY89714)), known COD genes (number 8: *Vibrio alginolyticus* H-8 (BAB21759), 9: *Vibrio parahaemolyticus* KN1699 (BAG70715), 10: *Vibrio* sp. SN84 (BAG82921), 11: *Vibrio cholerae O*1 (AAF94439) and 12: *Shewanella woodyi* ATCC 51908 (ACA84860)), chitin deacetylases (number 7: *Metagenomic* CDA (AEJ31921), 13: *Schizosaccharomyces pombe* (CAB10114), 14: *Colletotrichum Lindemuthianum* (2IW0), 15: *Steptomyces coelicolor* A3(2), (NP_630347) and 16: *Bacillus Lichenformis* (AAU22686)) and an allantoinase analog PuuE (number 17: *Pseudomonas fluorescens*, *puuE* (ACA50280)). Values above the diagonal line refers to percent sequence identity and values below the diagonal refer to number of differences.

COD genes were found in six strains and in both families. The COD genes were pairwise compared to other known COD genes and chitin deacetylases of bacterial and fungal origin. We included allontoinase genes since BLASTp analysis revealed that the *Vibrio* COD genes had 99% sequence similarity to allantoinases. Allantionases are enzymes that catalyze the hydrolytic cleavage of the hydantoin ring in allantoin, which is present in purine-derived compounds [29]. *Vibrio* COD genes had 96%–99% sequence similarity to chitin deacetylases (CDA) and the *Pseudoalteromonas* COD genes had 98%–99% sequence similarity to polysaccharide deacetylases (Figure 1). The *Vibrio* COD genes only had a low (10%–14%) similarity to other known COD genes from same genus. However, they share approximately 53% similarity with a chitin deacetylase, which was cloned from a metagenomic sample. The *Pseudoalteromonas* CODs had low similarity to the known CODs and to the metagenomic chitin deacetylase, 10% and 24%, respectively. The putative COD genes in our study were 10% similar to a *Shewanella* COD, which had more than 60% similarity to the known COD genes from *Vibrio* species. Low similarity was also found when comparing to fungal CDA. The Vibrio COD genes had low similarity to allantoinases from *Streptomyces coelicolor* and *Bacillus lichenformis*, however approximately 69% similarity to an allantoinase analog (PuuE) from *Pseudomonas fluorescens*.

#### 2.1.1. Phylogenetic Analysis of Chitinases

**Figure 2:**
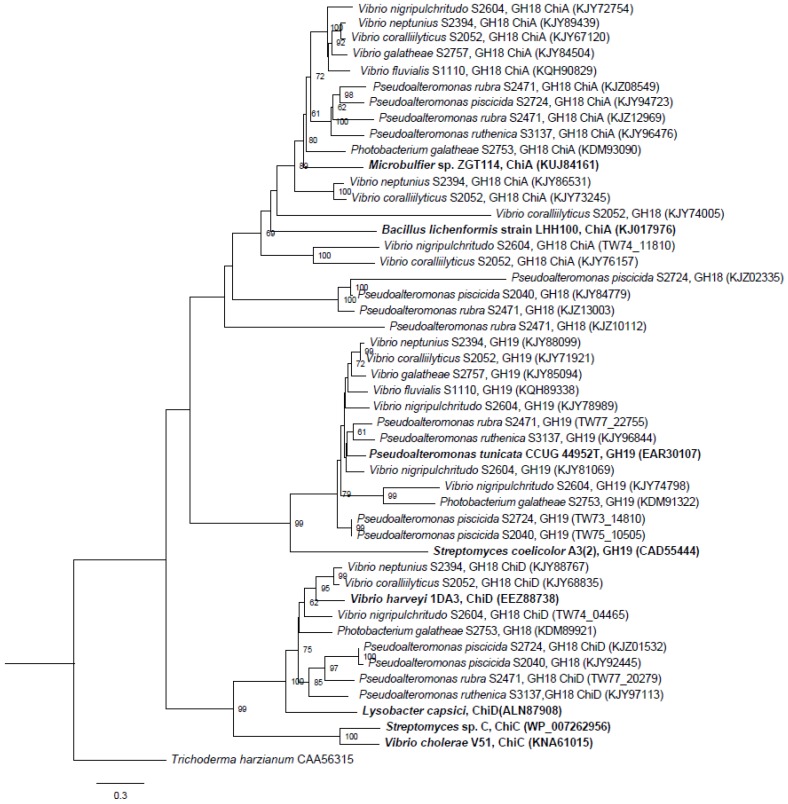
Phylogenetic relationship of the RAST-annotated chitinases from this study and known chitinases from the NCBI database (marked in bold). Branch support values (bootstrap proportions, with 1000 replicates in the analysis) are associated with nodes indicating that the support was <50%. The bar marker indicates the number of amino acid substitutions. Identifiers include species name and GH family, subfamily and accession numbers.

A phylogenetic tree comparing all the complete, translated chitinase genes identified in the 10 chitinolytic strains with other known chitinase genes from the GH18 and GH19 families was constructed. The genes clearly clustered within their respective families GH18 ChiA, GH18 ChiD and GH19, and, thus, were correctly annotated (Figure 2). The unclassified GH18 chitinases did not cluster with any known chitinases. The unclassified GH18 chitinase genes clustered together, with the exception of KJZ10112 from *P. rubra* S2471 that clustered alone, suggesting a new chitinase group.

**Figure 3:**
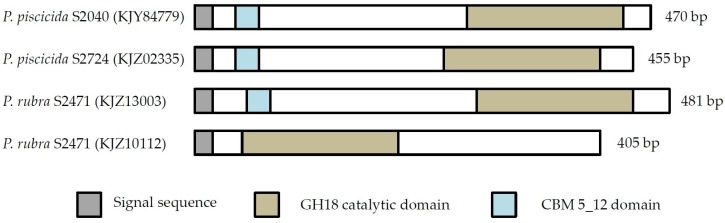
Domain structure of unclassified GH18 chitinases. Protein domains, as identified in Pfam, are shown. Blank areas in part of the proteins indicate that no match with characterized protein domains were found.

The unclassified GH18 chitinases contained signal peptides and catalytic GH18 domains (Figure 3). Three of four unclassified chitinases also contained a chitin binding motif (CBM). These domains provide evidence for their classification as chitinases.

#### 2.1.2. Cloning of Chitinases from S2753 and S2724

**Figure 4:**
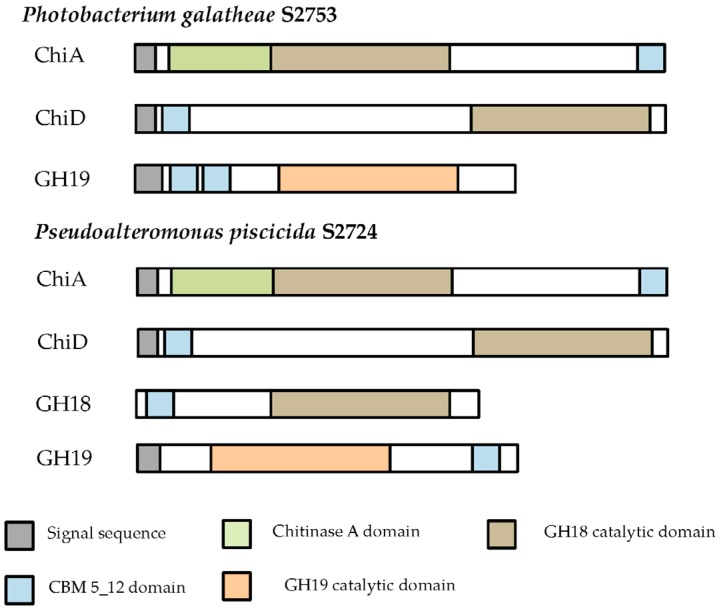
Domain structure of the cloned chitinases. Protein domains, as identified in Pfam, are shown. Blank areas in part of the proteins indicate that no match with characterized protein domains were found.

Chitinases from two strains with pronounced chitin degradation were cloned into a pBAD_His vector and expressed in an *E. coli* BL21 (DE3) host (Table 2). Chitinases are extracellular enzymes since a signalP detected signal peptides was found in all chitinases, as seen in the protein domain structure (Figure 4). The GH18 chitinase from S2724 (KJZ02335) was cloned without its signal peptide, as this region was left out from the RAST annotation.

**Figure 5:**
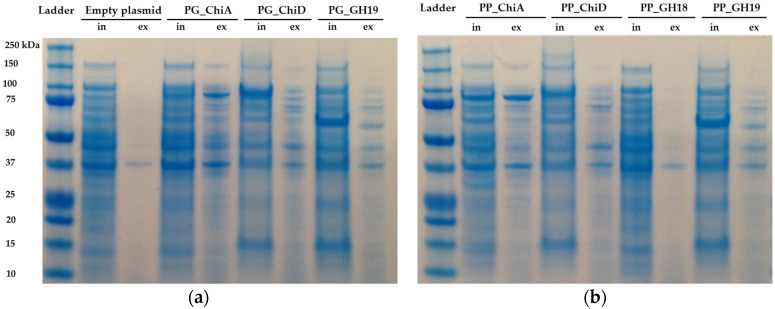
SDS-PAGE of induced *E. coli* BL21 harboring the cloned chitinases from (**a**) *P. galatheae* S2753 and (**b**) *P. piscicida* S2724 including both intracellular proteins (in) and extracellular protein extracts (ex). Expected protein sizes are PG_ChiA: 88 kDa, PG_ChiD: 88 kDa, PG_GH19: 59 kDa, PP_ChiA: 88 kDa, PP_ChiD: 90 kDa, PP_GH18: 49 kDa and PP_GH19: 53 kDa.

SDS-page analysis was used to determine if the chitinases were secreted or accumulated inside the cells. PG_ChiA and PP_ChiA were the only enzymes secreted (Figure 5).

**Figure 6:**
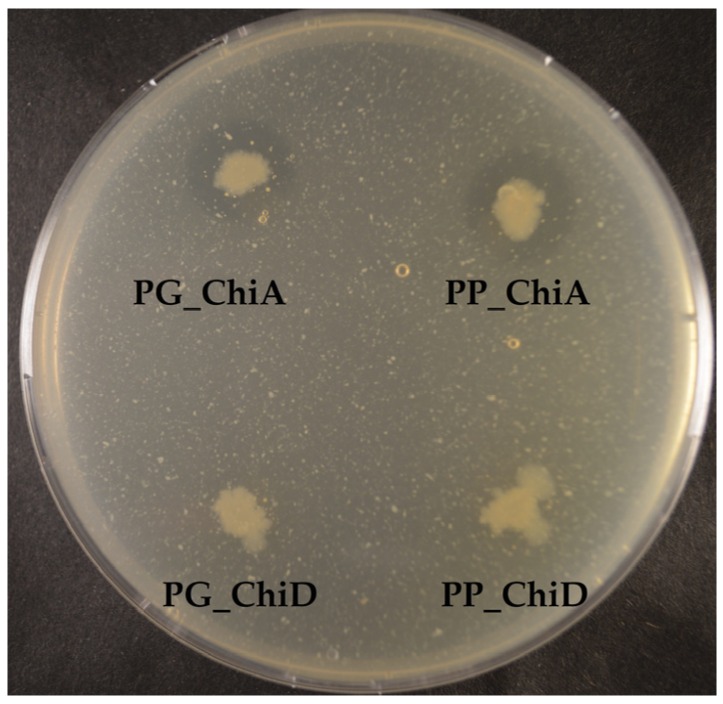
Colloidal chitin degradation by *E. coli* BL21 (DE3) expressing chitinase genes. The two ChiA-type chitinases are secreted, whereas the two ChiD-type chitinases accumulate inside the cells.

To test the substrate specificities of the chitinases, we tested the intracellular and extracellular extracts as well as the induced actively growing *E. coli* clones on crystalline and colloidal chitin and chitosan. Only ChiA clones and extracts degraded colloidal chitin but not crystalline chitin and not chitosan (Figure 6). Since we tested clone extracts, the protein concentrations of the chitinases were not determined, as other proteins are also present in the extracts.

#### 2.1.3. Antifungal Activity

**Figure 7:**
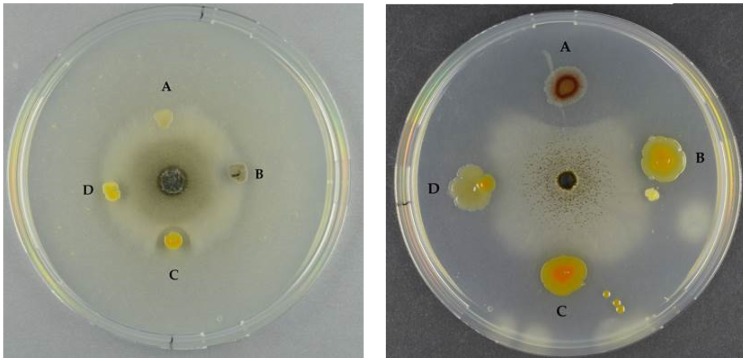
**Left panel**: Antifungal assay of *S. chartarum* by (**A**) S3137 (no inhibition); (**B**) S3431 (inhibition); (**C**) S2040 (inhibition); and (**D**) S2724 (inhibition). **Right panel**: *A. niger* and (**A**) S2471 (inhibition); (**B**) S2724 (inhibition); (**C**) S2040 (inhibition); and (**D**) S3137 (inhibition). Left panel bacteria were spotted after fungal inoculation, right panel bacteria were spotted prior to fungal inoculation.

Some chitinases have antifungal activity, and we tested the extracts from the chitinase clones against seven different fungi. None of the extracts were antifungal. We also tested the wild-type marine bacteria in two settings: one where the bacteria were spotted after fungal inoculation and one where the bacteria were spotted prior to fungal inoculation (Figure 7).

In the first setting, one strain, S2040, had a pronounced antifungal effect against all seven fungi (Table 3, columns A). The remaining 10 strains were antifungal against *A. niger* and *B. cinerea*, however, they were not capable of retaining the antifungal effect over time. In the second setting (where the bacteria were spotted prior to the fungi), the same scenario as described above occurred. In addition, the two fungi with the slowest growth rates, *S. chartarum* and *B. cinerea* were inhibited by the bacterial presence (Table 3, columns P). In this setting, two additional bacteria, S3137 and S2471, retained their antifungal activity over time against *P. chrysogenum* and *A. niger*, respectively.

## 3. Discussion

Chitin degradation is an important process in both marine and terrestrial environments. Chitin is also an important resource in different industrial and medical processes, and chitin degrading enzymes or microorganisms are of interest, e.g., as antifungal agents or as bio-insecticides. Hence, there is a growing demand for new enzymes with chitin-modifying properties. We analyzed 11 marine bacteria with antibacterial activity and found a remarkable potential for chitin degradation. In silico, we identified genes involved in the chitin degradation process using an iterative strategy. We found a total of 50 putative chitinase genes of which 11 were considered to be wrongly annotated. The strains contained from two to six putative chitinase genes, and the number of chitinase genes per strain did not correlate with phenotypic chitin degradation ability. The three strains causing the largest clearing zones in chitin agar, S2753, S2724 and S2040, harbored three, three and four chitinase genes, respectively, whereas the poorer chitin degraders, S2050 and S2604, both had six chitinase genes. The chitinases were grouped in different categories of ChiA, ChiD and unclassified belonging to GH18 and GH19 chitinases.

Chitinolytic bacteria harbor different chitinases, most likely due to a specificity for different substrates [16,30,31,32]. Here, all but one bacterial strain contained at least one chitinase belonging to GH18 ChiA, whereas GH18 ChiD and unclassified GH18 chitinases were only present in some strains. This is not surprising, since ChiA-type chitinases are the most dominant chitinases in bacteria and play a key role in chitin degradation [16,33,34]. Also, this was the dominant chitinase group found in un-cultured bacteria [35]. *P. piscicida* S2040 does not encode for a ChiA-like chitinase, and yet was one of the most potent chitin degraders. The genome of S2040 contained one chitinase of the ChiD-like type, one belonging to GH19 and one unclassified GH18 chitinase, and chitinases of the ChiA-like type are not responsible for the pronounced chitin degradation by this strain. Four chitinases from strains S2724, S2040 and S2471 did not cluster into any existing chitinase subgroups, suggesting they belong to a new subgroup of chitinases. The domain structure provides further evidence that these are indeed chitinases, as they contain the main characteristics of a chitinase (signal peptide, CBM and catalytic domain). However, one chitinase (KJZ10112) does not contain a CBM. This does not disqualify its classification as a chitinase. CBMs are important for the overall performance of the enzyme, but chitinases do not lose function without CBMs, they merely display weaker binding [36,37,38]. These four chitinases were only identified in the *Pseudoalteromonadaceae* family, and it is interesting to note that two of the chitinases, KJZ02335 and KJY84779, originate from two of the three most potent chitin degrading strains. These unknown chitinases may play a significant role in chitin degradation by *Pseudoalteromonas* species.

All strains had at least one gene encoding chitinases belonging to the GH19 family, which for many years were considered to be unique to higher plants. The first bacterial GH19 chitinase was found in 1996 [39] and the majority of GH19 chitinases have been found in *Streptomyces* species [14]. However, GH19 chitinases from *Vibrio*, *Aeromonas*, *Pseudoalteromonas*, *Chitiniphilus*, *Nocardiopsis* and *Burkholderia* species have also been described [8,40,41,42,43,44]. The presence of the GH19 chitinase in all 10 chitinolytic strains could indicate that this gene is more widespread in the marine environment than hitherto believed.

Since the discovery of the function of LPMOs as facilitators of chitin degradation, the interest in these particular enzymes has increased. Our in silico analysis revealed that LPMOs are present in nine of the 10 genomes from chitinolytic bacteria. Recombinant LPMOs have not yet been characterized in marine bacteria and this potential should be further explored. These LPMOs could be of interest in enzyme cocktails as shown in a recent study where a LPMO from a *Streptomyces griseus* increased the chitin solubilization yields by up to 30-fold when combined with a *Serratia marcescens* GH18 chitinase [45].

COD genes have so far only been identified in *Vibrio* species and in *Shewanella woodyi* ATCC51908 [46,47]. Here, we found six putative COD genes, however, they only had little homology to known CODs. The *Vibrio* COD genes were similar to the allantoinase analog PuuE indicating a wrong annotation. Additional evidence for the wrong annotation is the observation that the neighboring genes to the putative *Vibrio* CODs are involved in purine degradation. PuuE type allantoinases have high similarity to polysaccharide deacetylases, which is also the observation in this study, where the *Vibrio* COD genes had approximately 50% identity to a CDA from a metagenomic sample [48]. The putative COD genes from the two *Pseudoalteromonas* strains were only 23%–27% identical to the metagenomic CDA and the PuuE allantoinase. PuuE proteins can be distinguished from polysaccharide deacetylases by two highly conserved segments, which are only present in PuuE proteins. These conserved segments are present in the *Vibrio* genes (data not shown), but not in the *Pseudoalteromonas* genes, which provide further evidence for the classification of *Vibrio* CODs as PuuE type allantoinases. In contrast to the *Vibrio* COD genes, the *Pseudoalteromonas* COD genes have signal peptides. Thus, these CODs are likely secreted, and they may therefore potentially act as CDAs. CDAs of bacterial origin are of particular industrial interest, as they catalyze the conversion of chitin to chitosan, a highly coveted polymer [49]. Irrespective of the real function of the so-called *Pseudoalteromonas* COD, the enzymatic deacetylation of polymers has a high industrial potential.

Two of the seven cloned chitinases were secreted whereas the remaining five accumulated inside the cells. The signal peptide of ChiA type chitinases can be recognized by the secretion system in *E. coli*, and hence it is not surprising that ChiA is secreted by *E. coli.* [50,51]. However, cloned ChiA type chitinases are not always secreted [52], so the secretion does not seem to be specific to the chitinase type, but the secretion of the two ChiA-type chitinases from this study allows for an easy purification and exemplifies the potential industrial use of these enzymes. Extracellular and intracellular extracts were tested on different chitinous media, but only the extracts of the two ChiA enzymes degraded colloidal chitin, which may be due to substrate specificity and/or need for synergy between chitinases, as already mentioned.

We tested the extracts against seven fungi, covering both indoor contaminants and plant pathogens. GH19 chitinases are thought to be the main antifungal chitinases, but ChiA type chitinases have also displayed antifungal activity, like *Stm*ChiA from *Stenotrophomonas maltophilia* which was antifungal against *F. oxysporum* [52]. However, extracts from ChiA clones were not antifungal, nor were any of the other extracts. Since chitinase concentration in the extracts was unknown, an up-concentration could potentially result in a measurable effect.

All 11 bacterial strains, including the non-chitin degrader, were antifungal when tested as live cultures, however, with different fungal targets. Allowing pre-growth of the potential producer prior to fungal inoculation increased the antifungal activity of some of the bacteria, which is in agreement with the study by Giubergia and co-workers [27], in which bioactivity of a collection of *Vibrionaceae* increased 3-fold when the producer was allowed a 2-day pre-growth period. *P. piscicida* S2040 displayed pronounced antifungal effect towards all fungi, independently of the time of spotting, and hence would serve as a candidate for a broad-range antifungal bio-pesticide.

In summary, three of the ten strains were of interest due to their remarkable chitin degrading abilities and their antifungal activities. These strains could have potential in biodegradation of chitin-waste, and in biocontrol of unwanted fungal growth in agriculture and the building industry.

## 4. Materials and Methods

### 4.1. Strains and Plasmids

The bacterial strains used in this study (Table 4).

The bacteria were isolated during the Galathea 3 expedition [25] and they have been whole-genome sequenced [28]. Genomes were assembled using CLC Genomics Workbench 7 (CLC bio, Aarhus, Denmark) and contig-based draft genomes were obtained. Gene annotation was performed using Rapid Annotation using Subsystem Technology (RAST), [53,54,55]. The genomes are available at the National Center for Biotechnology Information (NCBI) [56]. To compare annotations, the genomes were also downloaded from NCBI containing the NCBI-annotated genes. *Escherichia coli* Top10 was used for cloning and propagation of plasmids, and *E. coli* BL21 (DE3) was used for expression of chitinase genes from *P*. *galatheae* S2753 and *P. piscicida* S2724. The cloning and expression vector was pBAD_Myc_HisA. Plasmids were isolated using the QIAprep^®^ Spin Miniprep kit (Qiagen, 27106, Hilden, Germany), and genomic DNA was extracted using the NucleoSpin^®^ Tissue kit (Machery-Nagel, 740952, Düren, Germany). Strains and plasmids used for cloning can be seen in Table 5.

### 4.2. Preparation of Colloidal Chitin

Colloidal chitin was prepared from shrimp shell chitin (Sigma, C7170, Deisenhofen, Germany) or crab shell (Sigma, C9752, Deisenhofen, Germany) chitin as follows: 10 g chitin was hydrolyzed in 400 mL ice-cold 37% HCl for 6 h at 4 °C with stirring. The solution was transferred to 4 L cold dH_2_O over night for settlement of chitin. The solution was neutralized using NaOH and adjusted to pH 7. Colloidal chitin was collected by centrifugation at 4000× *g* for 5 min and resuspended in dH_2_O for a final concentration of 2%. The chitin solution was autoclaved at 121 °C for 15 min.

### 4.3. Chitinase and Chitosanase Activity Screening

The strains were tested for chitinase and chitosanase activity on plates containing different chitinous substrates. The basic media consisted of 2% Sea Salt (Sigma, S9883, Deisenhofen, Germany), 1,5% agar, 0.3% casamino acids and was supplemented with either 0.2% colloidal chitin from shrimp or crab, 0.2% crystalline shrimp chitin or 0.2% shrimp chitosan (Sigma, 50494, Deisenhofen, Germany) or crab chitosan (Sigma, 48165, Deisenhofen, Germany). Plates were spotted with one single colony from a streaked culture from freeze-stock on marine agar plates (Difco 2216). The plates were incubated at 4, 15, 25 and 35 °C for 11 days for colloidal chitin and chitosan and 35 days for crystalline chitin. The natural turbidity of the media allows for visual evaluation of chitin/chitosan degradation appearing as a clearing zone around the spotted bacteria. A qualitative grading of chitinase activity (clearing zone) was given, determined from the edge of the bacterial colony to the edge of the clearing zone, where a zone of 0–6.99 mm was graded one plus, +, and >7 mm was graded two plusses, ++.

### 4.4. In Silico Analysis

An annotation based search for chosen genes involved in chitin degradation was conducted using CLC Main Workbench 7 and included genes putatively encoding chitinases, chitin sensors (ChiS or CdsS), COD and LPMOs. 50 chitinase-encoding genes were found using RAST. The complete genes were translated to protein, and divided into GH18 and GH19 families by analysis using the Pfam protein family database [57,58] and further divided into subfamilies by a protein blast in the non-redundant database in NCBI [59]. Since chitinases are extracellular proteins they were checked for signal peptides using SignalP 4.1 [60]. 11 RAST-annotated chitinases were eliminated from the analysis, as they did not contain either GH18 or GH19 domains, and two others were deleted as they did not contain signal peptides or CBMs. All included and eliminated chitinases can be seen in Supplementary Materials Table S1. Since the COD proteins showed little homology to other known CODs, they were blasted against the non-redundant protein database to find proteins of high sequence similarity. The COD encoding genes were compared to already known CODs, chitin deacetylases and an allantoinase analog by pairwise comparison in CLC.

### 4.5. Phylogenetic Analysis of Chitinases

A phylogenetic tree was created consisting of the identified chitinase genes from the ten chitinolytic strains. To indicate correct groupings and potentially group the unidentified chitinase genes, known chitinase genes belonging to the GH18 subfamilies A, C and D and GH19 chitinases from the NCBI database were included. The phylogenetic tree was created from a multiple alignment of the translated chitinase proteins with a neighbor-joining construction method and a bootstrap analysis with 1000 replicates was included. The tree was visualized using FigTree [61]. A fungal chitinase from *Trichoderma harzianum* (accession number CAA56315) was used as root.

### 4.6. Construction of Plasmids for Chitinase Expression

Expression plasmids for the seven chitinase genes from *P. galatheae S2753* and *P. piscicida* S2724 were constructed via USER cloning, as preciously described [62], using pBAD-Myc_HisA as the vector plasmid. Plasmid and chitinase genes were amplified by PCR using PfuX7 polymerase [63] and primers (Table 6) using following settings: initial denaturation at 98 °C for 2 min, 30 cycles of 98 °C for 20 s, 57 °C for 20 s, 72 °C for 1:30 min and a final extension at 72 °C of 2:30 min. For PG_GH19, PP_GH18 and PP_GH19 extension time was reduced to 50 s and final extension time reduced to 1:45 min. For plasmid DNA, extension time was prolonged to 2:30 min and final extension prolonged to 4:30 min. Each PCR reaction (50 μL) consisted of 5 μL Pfu buffer (200 mM Tris-HCl pH 8.8, 100 mM KCl, 60 mM (NH_4_)_2_SO_4_, 20 mM MgSO_4_, 1 mg/mL BSA (in nuclease-free water) and 1% Triton X-100), 5 μL dNTPs (2 mM), 1.2 μL MgCl_2_ (50 mM), 5 μL forward primer (5 μM), 5 μL reverse primer (5 μM), 1 μL PfuX7 polymerase and 0.5–1 μL DNA.

In short, the chitinase containing plasmids were constructed in a reaction of 10 μL, consisting of 100 ng of each purified PCR product, 1 μL T4 DNA ligase buffer (New England BioLabs, #B0202S, Ipswich, MA, USA ) and 1 μL USER™ enzyme (New England BioLabs, #M5505S, Ipswich, MA, USA). The reaction was incubated at 37 °C for 15 min, followed by 26 °C for 15 min and 10 °C for 10 min. 3 μL USER reaction was mixed with 40 μL chemically competent *E. coli* Top10. The mixture was incubated on ice for 30 min, followed by a 60 s heat shock at 42 °C, and 2 min incubation on ice. Cells were recovered in 1 mL LB for 1 h and subsequently harvested at 1600× *g* for 2 min. The pellet was plated on LB agar containing 100 μg/mL ampicillin and incubated at 37 °C overnight, and the following day colonies were grown in LB media with antibiotics and stored as glycerol stocks. Plasmids were purified and confirmed by sequencing (Macrogen, Amsterdam, The Netherlands).

### 4.7. Protein Expression and SDS-Page Analysis

Protein expression was initiated by transformation of the purified plasmids into electrocompetent *E. coli* BL21 (DE3) cells. Transformants were incubated at 37 °C overnight and one colony was used to incubate 10 mL LB media supplemented with 100 μg/mL ampicillin and incubated at 37 °C, 250 rpm, overnight. ON culture was used to incubate fresh LB media supplemented with ampicillin. At OD_600_ between 0.5 and 0.8 the cultures were induced with arabinose (total concentration 0.02%) and the cultures were further incubated at 37 °C for 4 h. 2 mL cell suspension was centrifuged for 1 min at 12,000× *g*. The supernatant was separated from the pellet, and the pellet was resuspended in 1 mL lysis buffer (50 mM potassium phosphate, pH 7.4, 500 mM sodium acetate, 0.1 mM EDTA and 20% glycerol). The lysis mixtures were kept on ice and lysed by sonication (Soniprep, amplitude 10 μm) for 2 × 30 s, and hereafter centrifuged for 1 min at 12,000× *g*. For SDS-page, protein concentration was estimated using the method of Bradford [64] with BSA as standard. Protein concentration of extracellular extracts ranged from 0.1 to 0.3 mg/mL and intracellular were diluted to a total concentration of 0.5 mg/mL. Fifty μL of protein extract were incubated with 10 μL loading dye (300 mM Tris HCl, pH 6.8, 0.01% bromophenol blue, 15% *v*/*v* glycerol and 6% SDS) at 95 °C for 5 min and 20 μL of each solution was loaded on a precast 4%–12% Bis-Tris gel (NuPAGE™ Novex™, Thermo Scientific, NP0321, Waltham, MA, USA). The gel was run for 1.5 h at 90 V and stained with Coomassie Brilliant Blue G-250. To identify the proteins on the gel, the sizes were estimated using the compute PI webtool [65].

### 4.8. Antifungal Activity

Seven different fungi were used for antifungal activity testing of both wildtype bacteria as well as the cloned chitinase enzyme extracts. The following fungi were chosen to cover indoor mold and plant-pathogenic fungi: *Penicillium chrysogenum* (IBT 33843), *Stachybotrys chartarum* (IBT 7709), *Chaetomium globosum* (IBT 7029), *Neosartorya hiratsukae* (IBT 28630), *Aspergillus niger* (IBT 32191), *Fusarium oxysporum* (IBT 41964) and *Botrytis cinerea* (IBT 41856) and were from the IBT Culture Collection at Department of Biotechnology and Biomedicine, Technical University of Denmark. The antifungal activity of wildtype bacterial strains was tested by adding a 20 μL spore suspension to a puncture well on the center of a MA plate. Due to different growth rates, the fungi were allowed to grow for 4–8 days, after which colony mass of each wildtype bacteria was spotted approximately 2 mm from the edge of the fungal colony. The plates were left for 4 days and antifungal activity was observed. We also tested the antifungal activity of the wild-type strains in a setting where the bacteria were spotted 2 days prior to the inoculating the fungi. The bacteria were spotted 2 cm from center of the plates. In both the above settings, plates were checked again after approximately 14 days to see if the bacteria retained their inhibitive effect. Antifungal effect was graded qualitatively, where one plus (+) describes antifungal effect which did not retain the effect after 14 days, and two pluses (++) describes the antifungal effect which was retained after 14 days.

For the chitinase extracts, the fungi were inoculated as described above and after 3–4 days of growth, holes were punched 2 mm from the edge of the fungal colony and 50 μL of each enzyme solution, prepared as in Section 4.7, was added to the wells. Plates were incubated for 2–4 days and checked for antifungal activity. All experiments were conducted at room temperature.

## Figures and Tables

**Table 1:** Chitinolytic activity and chitinase genes in 11 marine bacteria. Strain S3431 is included as a non-chitin degrading control. Chitinase activity was graded according to clearing zone size (+ is zone size of 0–6.99 mm, ++ is zone size of >7 mm and − is no clearing zone). Activity was evaluated at 25 °C on colloidal shrimp chitin. Chitinases can be grouped into glycosyl hydrolases (GH) families 18 and 19. GH18 chitinases are further sub-grouped into ChiA, ChiD and unspecified (U).

Strain	Species	Chitinase Activity	# Of Chitinolytic Enzymes						CSS Type
			GH18 (ChiA)	GH18 (ChiD)	GH18 (U)	GH19	COD	LPMO	
S2753	*P. galatheae*	++	1	1 *	0	1	0	1	ChiS
S2052	*V. coralliilyticus*	+	4 **	1	0	1	1	2	ChiS
S2604	*V. nigripulchritudo*	+	2	1	0	3	1	1	ChiS
S2394	*V. neptunius*	+	2	1	0	1	1	2	ChiS
S2757	*V. galatheae*	+	1	0	0	1	1	0	ChiS
S1110	*V. fluvialis*	+	1	0	0	1	0	1	ChiS
S2040	*P. piscicida*	++	0	1 *	1	1	1	1	CdsS
S2724	*P. piscicida*	++	1	1	1	1	1	2	CdsS
S3137	*P. ruthenica*	+	1	1	0	1	0	1	CdsS
S2471	*P. rubra*	+	2	1	2	1	0	2	CdsS
S3431	*P. fuliginea*	−	0	0	0	0	0	0	CdsS

CSS: chitin sensing system, ChiS: chitin catabolic cascade sensor histidine kinase in *Vibrionaceae*, COD: chitooligosaccharide deacetylase, CdsS: chitin sensor kinase in Pseudoalteromonas, LPMO: lytic polysaccharide monooxygenases; * Have been classified as ChiD, due to phylogeny, ** One chitinase has been classified as ChiA, due to phylogeny.

**Table 2:** Name, length and expected protein sizes of the cloned chitinases.

Protein	Name	Length (Amino Acids)	Expected Size (kDa)
KJY84504	PG_ChiA	834	87.7
KDM89921	PG_ChiD	846	87.9
KJY85094	PG_GH19	539	59.4
KJY94723	PP_ChiA	822	87.5
KJZ01532	PP_ChiD	850	90.4
KJZ02335	PP_GH18	455	49.3
TW73_14810 *	PP_GH19	479	53.1

* Annotated as pseudo-gene in the NCBI database.

**Table 3:** Inhibition of fungi by 11 marine bacteria. Bacteria were spotted after (A) or prior (P) to inoculation of fungi. (+) describes an initial antifungal effect which was not retained after 14 days and (++) describes an antifungal effect which was retained after 14 days. (−) no antifungal effect, (sg) sparse growth of the fungi, (nt) not tested.

Strain	Activity against Fungal Strains													
	*Penicillium chrysogenum*		*Stachybotrys chartarum*		*Chaetomium globosum*		*Neosartorya hiratsukae*		*Aspergillus niger*		*Fusarium oxysporum*		*Botrytis cinerea*	
	A	P	A	P	A	P	A	P	A	P	A	P	A	P
S2753	+	nt	+	sg	−	nt	+	nt	+	nt	+	nt	+	sg
S2052	+	+	+	sg	−	−	−	+	+	+	−	−	+	sg
S2604	−	+	−	sg	−	+	+	+	+	+	−	−	+	sg
S2394	−	nt	−	sg	−	nt	−	nt	+	nt	−	nt	+	sg
S2757	−	−	−	sg	−	+	−	−	+	−	−	−	+	sg
S1110	+	+	−	sg	−	−	−	−	+	+	−	−	+	sg
S2040	++	++	++	sg	++	++	++	++	++	++	++	++	++	sg
S2724	+	+	+	sg	−	−	−	−	+	+	−	−	+	sg
S3137	+	++	−	sg	−	+	+	+	+	+	−	+	+	sg
S2471	+	−	−	sg	−	+	−	−	+	++	−	−	+	sg
S3431	+	nt	+	nt	−	nt	−	nt	+	nt	−	nt	+	sg

**Table 4:** Marine strains used in this study.

Strain	Species	Accession Number
S2753	*Photobacterium galatheae*	JMIB01
S2052	*Vibrio coralliilyticus*	JXXR01
S2604	*Vibrio nigripulchritudo*	JXXT01
S2394	*Vibrio neptunius*	JXXU01
S2757	*Vibrio galatheae*	JXXV01
S1110	*Vibrio fluvialis*	LKHR01
S2040	*Pseudoalteromonas piscicida*	JXXW01
S2724	*Pseudoalteromonas piscicida*	JXXX01
S3137	*Pseudoalteromonas ruthenica*	JXXZ01
S2471	*Pseudoalteromonas rubra*	JXYA01
S3431	*Pseudoalteromonas fuliginea*	JJNY01

**Table 5:** Cloning and expression hosts and plasmids used in this study.

Strain/Plasmid	Details	Reference
*Escherichia coli* Top10	Cloning host	Invitrogen, C404010, Paisley, United Kingdom
*Escherichia coli* BL21 (DE3)	Expression host	Novagen, Madison, WI, USA
pBAD_Myc_HisA	Cloning and expression vector	Thermo Scientific, V44001, Waltham, MA, USA
PG_ChiA	pBAD_Myc_HisA vector containing the EA58_02560 gene	This study
PG_ChiD	pBAD_Myc_HisA vector containing the EA58_19900 gene	This study
PG_GH19	pBAD_Myc_HisA vector containing the EA58_12180 gene	This study
PP_ChiA	pBAD_Myc_HisA vector containing the TW73_17595 gene	This study
PP_ChiD	pBAD_Myc_HisA vector containing the TW73_14030 gene	This study
PP_GH18	pBAD_Myc_HisA vector containing the TW73_13265 gene	This study
PP_GH19	pBAD_Myc_HisA vector containing the TW73_14810 gene	This study

**Table 6:** Primers used for chitinase gene amplification.

Primer	Sequence 5′–3′
pBAD_Myc_HisA_fw	AATTCGAAGCUTGGGCCCGAA
pBAD_Myc_HisA_rv	ATGGTTAATUCCTCCTGTTAGCC
PG_ChiA_fw	AATTAACCAUGTCTTTCAATAAGTTGAGTCCTATTGC
PG_ChiA_rv	AGCTTCGAATUCTGGCAGTTTGCTGCACCCA
PG_ChiD_fw	AATTAACCAUGCGTAAAACTCTGATTCAGACAGCTGT
PG_ChiD_rv	AGCTTCGAATUCTGAGCGTTCATAGCATCCAGCTTC
PG_GH19_fw	AATTAACCAUGAAACAAAAACTGTCCCCTCAATGGG
PG_GH19_rv	AGCTTCGAATUCTCAACGGTGACACCATAATATTTCTGG
PP_ChiA_fw	AATTAACCAUGAAACTTAATAAAATAACCAGCTATATAGGACTTG
PP_ChiA_rv	AGCTTCGAATUGTTAGTTACTGCCTTCCATACATCAGC
PP_ChiD_fw	AATTAACCAUGAAACCAACTTCTATATTACGATTGGCTTGG
PP_ChiD_rv	AGCTTCGAATUATTTCCTTGATTCATCTGCGTTAATTTATCGC
PP_GH18_fw	AATTAACCAUGGAAGTTGCACTGGCGGTTGACT
PP_GH18_rv	AGCTTCGAATUCTGACATTGATAGCTTGGTGTTACACCA
PP_GH19_fw	AATTAACCAUGAACAGTCTAAAATTAGCGACCGCAGTT
PP_GH19_rv	AGCTTCGAATUGTTAACCGCTAACCAAGGACCCG

## Supplementary Materials

The following are available online at http://www.mdpi.com/1660-3397/14/12/230/s1, Table S1: List of included and excluded chitinases.
