# Supplementary material for: Biological Potential of Chitinolytic Marine Bacteria
Source: Mar Drugs. 2016 Dec 16;14(12):230. doi: 10.3390/md14120230 (PMC5192467; doi:10.3390/md14120230)
Supplement: Supplementary file 1 [file marinedrugs-14-00230-s001.docx]

Supplementary Materials: Biological Potential of Chitinolytic Marine Bacteria

Sara Skøtt Paulsen, Birgitte Andersen, Lone Gram and Henrique Machado

**Table S1.** List of included and excluded chitinases.

| **Locus-Tag** | **Protein ID** | **Length (aa)** | **GC-Content** | **GH Family** | **Signal Peptide** | **CBM** | **Included in Analysis** |
| --- | --- | --- | --- | --- | --- | --- | --- |
| EA58_02560 | KDM93090 | 833 | 52.1 | 18 | yes | 1 | yes |
| EA58_19900 | KDM89921 | 847 | 51.3 | 18 | yes | 1 | yes |
| EA58_12180 | KDM91322 | 539 | 50.2 | 19 | yes | 2 | yes |
| TW71_23380 | KJY67120 | 842 | 49.9 | 18 | yes | 1 | yes |
| TW71_19985 | KJY68835 | 847 | 51.1 | 18 | yes | 1 | yes |
| TW71_09905 | KJY74005 | 822 | 43.3 | 18 | yes | 1 | yes |
| TW71_13115 | KJY71921 | 551 | 49.6 | 19 | yes | 1 | yes |
| TW71_11440 | KJY72937 | 414 | 40.2 | NI | yes | 0 | no |
| TW71_05305 | KJY76755 | 872 | 48.9 | NI | yes | 2 | no |
| TW71_10410 | KJY73182 | 446 | 40.3 | NI | yes | 0 | no |
| TW71_10755 | KJY73245 | 851 | 47.7 | 18 | yes | 1 | yes |
| TW71_06185 | KJY76157 | 950 | 48.4 | 18 | yes | 1 | yes |
| TW74_20140 | KJY73651 | 956 | 49.6 | NI | yes | 1 | no |
| TW74_11810 | none * | 578 | 45.7 | 18 | yes | 1 | yes |
| TW74_04465 | none * | 818 | 51.2 | 18 | no | 1 | yes |
| TW74_21760 | KJY72754 | 846 | 47.9 | 18 | yes | 1 | yes |
| TW74_13165 | KJY77251 | 182 | 44.3 | NI | no | 2 | no |
| TW74_19045 | KJY74798 | 483 | 48.5 | 19 | yes | 0 | yes |
| TW74_09840 | KJY78989 | 549 | 48.3 | 19 | yes | 1 | yes |
| TW74_01915 | KJY81069 | 493 | 48.2 | 19 | yes | 0 | yes |
| TW84_13205 | KJY88767 | 847 | 49.4 | 18 | yes | 1 | yes |
| TW84_12630 | none * | 464 | 37.5 | NI | no | 0 | no |
| TW84_15460 | KJY88099 | 551 | 49.2 | 19 | yes | 1 | yes |
| TW84_22815 | KJY82689 | 866 | 49.0 | NI | yes | 2 | no |
| TW84_11910 | KJY89439 | 842 | 50.0 | 18 | yes ^A^ | 1 | yes |
| TW84_23545 | KJY80580 | 371 | 41.7 | NI | yes | 0 | no |
| TW84_19270 | KJY86531 | 851 | 49.3 | 18 | yes | 0 | yes |
| TW81_02960 | KJY84504 | 845 | 49.5 | 18 | yes ^A^ | 1 | yes |
| TW81_16550 | KJY81957 | 864 | 49.8 | NI | yes | 2 | no |
| TW81_01875 | KJY85094 | 555 | 49.2 | 19 | yes | 1 | yes |
| AMR75_13200 | KQH89338 | 556 | 53.2 | 19 | no | 1 | yes |
| AMR75_05075 | KQH90829 | 849 | 53.6 | 18 | yes | 1 | yes |
| TW75_01760 | KJY92445 | 850 | 47.4 | 18 | yes | 1 | yes |
| TW75_10505 | none * | 479 | 47.6 | 19 | yes | 1 | yes |
| TW75_20845 | KJY84779 | 470 | 47.0 | 18 | yes | 1 | yes |
| Tw75_04455 | KJY91643 | 280 | 43.2 | 18 | no | 0 | no |
| TW73_14030 | KJZ01532 | 850 | 47.0 | 18 | yes | 1 | yes |
| TW73_17595 | KJY94723 | 822 | 46.7 | 18 | yes | 1 | yes |
| TW73_13265 | KJZ02335 | 456 | 47.7 | 18 | yes ^A^ | 1 | yes |
| TW73_14810 | none * | 479 | 47.1 | 19 | yes | 1 | yes |
| TW72_16775 | KJY96476 | 871 | 51.7 | 18 | yes | 2 | yes |
| TW72_16685 | KJY96844 | 546 | 53.3 | 19 | yes | 2 | yes |
| TW72_14880 | KJY97113 | 849 | 51.6 | 18 | yes | 1 | yes |
| TW77_11900 | KJZ08549 | 821 | 50.9 | 18 | yes | 1 | yes |
| TW77_07675 | KJZ10112 | 405 | 50.4 | 18 | yes | 0 | yes |
| TW77_01045 | KJZ12969 | 880 | 51.8 | 18 | yes | 2 | yes |
| TW77_01310 | KJZ13003 | 470 | 51.2 | 18 | yes | 1 | yes |
| TW77_22755 | none * | 479 | 51.5 | 19 | yes | 1 | yes |
| TW77_08815 | KJZ09882 | 504 | 46.5 | NI | no | 0 | no |
| TW77_20279 | none * | 854 | 52.9 | 18 | yes | 1 | yes |

CBM: Carbohydrate binding module, NI: Not identified, * Annotated as pseudo-gene in the NCBI database, ^A^ Signal peptide not included in RAST annotation, however it is included in the NCBI annotation.
